# Feasibility of a text-messaging smoking cessation program for soldiers in Israel

**DOI:** 10.1186/s12889-019-6958-z

**Published:** 2019-06-26

**Authors:** Dov Bary-Weisberg, Marina Meltser, Maya Oberman, Avital Pato Benari, Yael Bar-Zeev, Sarit Shalev, Carla J. Berg, Lorien C. Abroms, Hagai Levine

**Affiliations:** 1 Braun School of Public Health and Community Medicine, Faculty of Medicine, Hadassah-Hebrew University, P.O Box 12272, Kiryat Hadassah, Ein Kerem, 9112002 Jerusalem, Israel; 2 Army Health Branch, Medical Corps, Israel Defense Forces, Tel Hashomer, Ramat Gan, Israel; 3 Israel Cancer Association, Givatayim, Israel; 4 Department of Behavioral Sciences and Health, Emory University Rollins School of Public Health, Atlanta, Georgia USA; 5 Department of Prevention and Community Health, Milken Institute School of Public Health, The George Washington University, Washington, District of Columbia USA

**Keywords:** Smoking cessation, Soldiers, Mhealth, Military, Digital health

## Abstract

**Background:**

Cigarette smoking is a main cause of preventable morbidity and mortality. Many young adults begin smoking in the military, with smoking rates higher among soldiers than in the general population. Among other health effects, smoking impairs performance among soldiers. Smoking cessation programs in the military are challenging due to the unique settings and low access to smoking cessation resources. Studies have shown that text-messaging smoking cessation programs are feasible and effective, but there is a lack of studies on soldiers.

**Objective:**

To evaluate the feasibility of a text-messaging smoking cessation program tailored for soldiers.

**Methods:**

We recruited 81 soldiers who smoked, 76.5% of whom were male. Following enrollment, participants filled out a baseline survey and were given a text messaging program for 6 months. Participants could send predetermined keywords and immediately receive a response from a list of messages that were constructed as a response to the specific keyword. Participants filled out a follow-up survey at 1 month. Additionally, we retrieved and analyzed program usage data, including keywords sent and received, for the entire program period. Based on the follow-up survey and the program usage data, we assessed feasibility of the recruitment methods, participants’ engagement and satisfaction and technical usability of the program.

**Results:**

At 1 month, 20.6% reported that they had not smoked in the past week. A high percentage of the participants were engaged in the program, with 82.5% sending at least one valid keyword. The lowest self-efficacy group had higher chances of leaving the program (50.0%) while for the highest group there were much lower chances (4.8%). Most of the soldiers (96.8%) found the program easy to use and would recommend it to a friend (84.1%).

**Conclusions:**

The study demonstrates that a text-messaging smoking cessation program is feasible in a military setting. Further development and evaluation of digital smoking cessation tools tailored for soldiers are warranted.

## Background

Tobacco use is the world’s largest preventable cause of death [1]. Epidemiological data suggest that smoking even less than one cigarette per day increases the risk of cancer and cardiovascular related diseases [2]. Among Israeli adults, 22.5% smoke cigarettes, with 24.8 and 14.9% of newly enlisted male and female soldiers being smokers, respectively [3]. Smoking affects the health of soldiers. Among soldiers who smoke, one study reported a significantly increased use of health care services and increased loss of training and active duty days in the Israeli military [4]. Others have reported higher rates of hospitalization among soldiers who smoke [5]. Roughly 18.4% of non-smoking young adults begin smoking during military service in Israel, and over 50% of former smokers relapse during military service, providing an important point of intervention that should not be overlooked [6].

Several studies have shown the benefit of text-messaging based smoking cessation interventions in helping users change their behavior. A randomized trial that included 5800 participants reported a doubling of biochemically verified continuous abstinence at 6 months for the intervention group (RR = 2.2, 95% CI [1.80–2.68]). The smoking status in this study was biochemically confirmed [7]. A meta-analysis that included five randomized control trials and a total of 8315 participants found participants to be twice as likely to report 7-day abstinence at 4 weeks (aOR = 2.89, 95% CI [2.57, 3.26]), and similar findings at 6 months in two of the studies (aOR = 2.24, 95% CI [1.90, 2.64]) [8]. A Cochrane review that included 12 studies conducted in high–income countries concluded that mobile-based programs, which were largely text messaging, resulted a 70% increase in chances of cessation. The six biochemically verified studies showed a significant advantage at 6 months compared to the control groups (RR = 1.83 95% CI [1.54, 2.19]; I^2^ = 71%; six studies; 7360 participants) [9]. Additional systematic reviews have found similar results [10, 11]. Furthermore, studies have found personalized-interactive smoking cessation programs via text-messaging to be acceptable among young adults [12]. Other studies have demonstrated success of such a program with sub-groups such as among veterans [13] and pregnant women [14, 15], supporting the idea that text-message based smoking cessation programs should be tailored to specific groups. A recent study [16] in Israel demonstrated the feasibility of iStopSmoke, which was adapted from the QuitNowTXT Message Library [17], a publicly-available version of the United States National Cancer Institute’s SmokefreeTXT program [18]. The adaptation process was similar to that described by Abroms et al. [19]. A full description for the iStopSmoke development and pilot study has been reported previously [16]. In brief, the development of iStopSmoke was based on the social cognitive theory [20], as part of a bio-behavioral model. The messages are built to improve the users’ self-efficacy for smoking cessation, highlight the consequences of continued smoking, and improve social support and the behavioral ability to quit smoking. Participants (*N* = 38), were Israeli smokers who spoke English. Surveys conducted 4 weeks after enrollment in the English language version of IStopSmoke assessed participant smoking status, technical issues and user satisfaction. Using an Intention-To-Treat (ITT) analysis, 23.7% of the participants reported that they had not smoked for 7 days. No participants reported technical issues receiving messages, and 23.3% reported technical issues sending responses or using keywords. Satisfaction rates were high, with 63.6% reporting that the program helped them quit smoking, and 68.2% stating they would recommend the program to a friend [16]. These results encouraged us to continue developing the program including a version in Hebrew.

Following the successful pilot study for iStopSmoke, we adapted a text-messaging smoking cessation program tailored for soldiers serving in the Israeli Defense Forces (IDF). We hypothesized that a program tailored specifically to the needs of this population would be an important addition to promoting smoking cessation among soldiers. The objective of this study was to evaluate whether the adapted program was feasible for soldiers in the IDF, including assessing recruitment methods, participants’ engagement and satisfaction and technical usability of the program.

## Methods

### Program development

Following the iStopSmoke pilot a Hebrew language program was developed for use in the military. The free-of-charge program was developed by a multi-disciplinary team and based on the experience and evidence supplied by the American SmokeFreeTXT and the Israeli iStopSmoke. Advice and comments were provided by military medical personnel and experts from the Israel Cancer Association, Ministry of Health, and the Israeli Medical Association for Smoking Cessation and Prevention as well as participant of iStopSmoke. The advisors reviewed text messages in the database and suggested revisions. Health professionals provided suggestions based on their clinical experience with smoking cessation, as well as specific experience with military personnel. Comments from the iStopSmoke program users were collected from the 2-week follow-up assessment survey filled out by the participants. Examples of changes to the program, following these suggestions are: 1) expanding the information provided through the program website 2) customization by gender. This was particularly important, because in addition to customizing for expected gender differences and appropriate recommendations, Hebrew has grammatical gender, requiring separate message formulation for men and women. While the general program was retained, several changes were made compared to iStopSmoke in order to tailor these to the unique culture and language in the military, and the aforementioned suggestions from iStopSmoke participants. Participants were offered to choose a quit day up to 14 days after registration instead of seven, and the entire program was extended to 6 months after the quit day instead of 30 days. Messages suggesting adopting a pet or going to the theater were replaced with recommendations relevant to the military setting, such as going for a run or reading a book. Relevant medical recourses within the military and civilian health systems in Israel were provided by text message throughout the program. Evaluation was updated, and used an online survey and phone survey as opposed to personal meetings.

Beginning at registration and up to the chosen quit day, the participants received between two and three daily messages. Participants received four daily messages during the first week after quit day, three daily messages for the next 2 weeks, and then two daily messages for the last week of the first month after quit day. During the second month, they received messages every other day, decreasing to weekly messages at day 90, and continuing in this manner until 180 days when the program was concluded. As in iStopSmoke the messages sent were relevant to the stage of the program, i.e. messages promoting cessation up until the quit date and messages encouraging the participants to remain smoke free. Examples for some of the messages can be found in Appendix 1. The participants were encouraged to interact with the program using the keywords described in Appendix 2. Examples of keywords included: NEED (when the participant felt the need to smoke), BOREDOM (for feeling bored), and NERVOUS (for feeling annoyed or anxious). For example, one of the possible responses to the keyword NEED was: “Try and think of other times you have overcome the need to smoke. What did you do? How did you feel after you succeeded?” Participants were provided periodic reminders of the possibility to send keywords throughout the program. Once a participant sent a keyword, he immediately received a response from a list of messages that were constructed as a response to the specific keyword. The program was conducted solely in Hebrew, including all text messages and surveys.

Following participant suggestions from our previous study [16], the program included a website that participants could access for additional information. The website included a description of the program, a detailed description of the keywords they could use, and a cigarette expense calculator. The technical support was provided by a private company, which implemented the program within an existing platform (Salesforce.com).

### Selection and recruitment of participants

The study was approved by the Hadassah Medical Center and IDF institutional review board (IRB) committees (approval number: 1490–2015). All participants provided consent by signing an electronic form and replying “approve” to a text message they received after completing the form.

Eligibility criteria for this study included: (1) being aged ≥18 years; (2) smoking at least one cigarette a day; (3) reporting willingness to try to quit smoking within 2 weeks; (4) having a cell phone with ability to send and receive text messages; (5) able to read and write Hebrew; and (6) currently serving in the IDF. Participants were excluded if they were pregnant. Participants were recruited from both “open” and “closed” units. In “open” units, soldiers do not sleep at their base, but rather sleep at home every night, similar to a day job. In “closed” units, they only go home for weekends (or less frequently).

Recruitment began on May 31, 2016 and ended August 11, 2016. Recruitment was promoted in several ways. Soldiers were contacted directly by sending e-mail messages out via the IDF internal e-mail system. In addition, doctors and clinic commanders received an e-mail encouraging them to promote the program among their patients. Mainstream media outlets published articles about the program. Public service announcements broadcasted on the IDF radio station encouraged soldiers to participate. Facebook ads were published, without “promotion”, through the public pages of the Israel Cancer Association and the IDF and posts were shown on the organization websites. The ad invited soldiers to join a smoking cessation text-message program tailored for their needs, free of charge as part of a study. Additionally, a short text message was sent to army doctors and soldiers encouraging them to pass it along to their friends and colleagues. Recent research has shown that social media in general and Facebook specifically can be useful methods of recruiting participants to health research studies [21].

The enrollment process had eight steps: (a) Participants completed a registration survey that could be reached through the Israel Cancer Association’s website or a direct link from the various digital advertisements. This survey was later served as their baseline survey if they were found eligible; (b) After completing the survey participants were immediately asked to choose a quit-date 2–14 days forward (c) Participants completed and electronically signed the consent form which appeared after completing the previous steps; (d) Participants were given the option to request that a member of the research team contact them before beginning the program (*n* = 29, 35.8%) (e) Upon submitting the form, participants received a text message requiring them to reply “accept” in order to finalize their registration for the study (and the program); (f) A member of the research time reviewed each participant to make sure they met the inclusion criteria. If requested, they called the participant and answered any questions; (g) The researcher approved the participant’s inclusion in the study and activated the program. Participants did not receive any financial compensation for participation and could withdraw from the program and/or the study at any point.

### Data collection

Participants completed a baseline survey, which was included in the consent form. The survey supplied data regarding sociodemographic characteristics (gender, age, country of birth, relationship status, education and base type), smoking characteristics (daily cigarettes smoked, past quit attempts, age of smoking initiation and time of first daily cigarette) and mobile phone usage. The full survey is included in Appendix 3.

Similar to previous research [15], usage data, reflecting engagement, was recorded in the program’s database. This included sent (keywords) and received text messages, as well as responses to messages regarding their smoking status throughout the program. The database included a precise date and time for all messages sent and received by each participant.

Participants received an online follow-up survey via email and text message 1 month after their chosen quit date. The question asked in the online survey regarding smoking status was: “Have you relapsed, and returned to smoking after quitting?” Those who answered yes to this question, were considered to still be smoking. In addition, a single question survey sent by text message asked: “Have you quit smoking since the beginning of the program?” This question was sent on days 7, 30, and 90, with low response rates of 36 (44.4%), 30 (37.0%), and 27 (33.3%) respectively. Due to low response rates and the question being unclear, we did not analyze the cessation rates. We analyzed the smoking cessation rates both as a percentage of the survey responders, and with a conservative estimate, assuming that all non-responders were still smoking (i.e., intent-to-treat analysis).

Participants who did not complete the online follow-up surveys were reminded once more by text message and then subsequently by phone where they were offered the option of completing the survey during the call. Up to five phone calls were made to each participant in order to receive as many surveys as possible. The participants completed the survey within 5 weeks. Participants who reported leaving the program were compared to those who remained among several variables, including sex and self-efficacy. Self-efficacy was defined on a five point Likert Scale (1-lowest, 5-highest), as the participant’s self-stated ability to quit smoking based on the answer to the question: “To what degree to do you rate your ability to quit smoking”, on the baseline survey. Technical usability was assessed by a user’s self-report of technical issues with the program during the study period.

### Data analysis

Baseline characteristics are presented as frequencies and percentages for the qualitative variables and means ± Standard deviation (SD) for the quantitative variables. Engagement was assessed by analyzing the use of keywords by the participants, number of participants that sent each of the keywords, proportion of participants who sent more than one keywords, and average number of keywords each participant sent. Additionally, the use of keywords was used as an indication of the program’s technical usability. The total number of keywords sent by the participants was also assessed from participant self-report, enabling assessment of both the subjective feeling of the participants in comparison to objective usage data.

Participant satisfaction and technical usability as reported in the survey are presented as number and proportion of participants who responded positively to the question. Variables were assessed for association with leaving the program using Chi-squared and Fisher’s exact test. Smoking status is presented as number of participants not smoking at follow-up and their proportion.

Feasibility was defined as a composite of previously described measures [22, 23]. It includes feasibility of the recruitment methods, participants’ engagement and satisfaction and technical usability of the program.

## Results

Eighty-five potential participants expressed interest in the program. Two of them were excluded for not being soldiers, and two more for not providing proper consent. Thus, 81 soldiers were enrolled in the study. The response rate for the online survey at 1 month follow up was 77.8% (*n* = 63/81). All participants texted in the keyword APPROVE, to complete their enrollment in the program. The 81 participants reported that they heard about the program through: E-mail (49.4%), Friends/family (22.2%), Clinic (9.9%), Facebook (9.9%), WhatsApp/SMS (3.7%), Commander (2.5%), Newspaper (1.2%), Website (1.2%).

### Participant baseline characteristics

The participants were mostly young (24.4 ± 8.2), single (*n* = 66, 81.5%) and male (*n* = 62, 76.5%). They reported smoking 13.0 ± 6.0 cigarettes daily, an average smoking initiation age of 16.7 ± 2.4 years, and several previous quit attempts (5.3 ± 8.1). Six (7.4%) reported smoking within 5 min of waking up in the morning. About half of the participants reported living with a smoker. They all owned a smartphone, and they reported sending 99.8 ± 209.0 text messages per week on average. 36 (44.4%) of the soldiers served in an “open” base, and the other 56.6% served in “closed” bases. (Table 1).

**Table 1:** Baseline characteristics of study participants (*N* = 81)

Sociodemographic characteristics		n (%) or mean ± SD
Gender	Female	19 (23.5)
	Male	62 (76.5)
Age	18–24, n (%)	57 (70.4)
	25–49, n (%)	23 (28.4)
	+ 50, n (%)	1 (1.2)
Country of birth, n (%)^a^	Israel	62 (76.5)
	Not Israel	18 (22.2)
Relationship status, n (%)	Single	66 (81.5)
	Married	15 (18.5)
	Divorced	0
Education, n (%)	More than a high school education	25 (30.9)
	Completed high school	55 (67.9)
	Less than a high school education	1 (1.2)
Base type	Open, n (%)	36 (44.4)
	Closed, n (%)	45 (55.6)
Smoking characteristics		n (%) or mean ± SD
Daily cigarettes smoked, mean ± SD		13.0 ± 6.0
Past quit attempts, mean ± SD		5.3 ± 8.1
Age of smoking initiation (years), mean ± SD		16.7 ± 2.4
Time of first daily cigarette, n (%)	Within 5 min	6 (7.4)
	5–30 min	24 (29.6)
	31–60 min	37 (45.7)
	Over 60 min	14 (17.3)
Presence of one or more smokers in household, n (%)		44 (54.3)
Days until chosen quit date, mean ± SD		10.5 ± 4.8
Mobile phone usage	Owns a smartphone, n (%)	81 (100)
	Number of texts sent per week, mean ± SD	99.8 ± 209.0

*NA* not available/applicable, *M* mean, *SD* Standard deviation

^a^Data is missing for one of the soldiers

### Program feasibility

A list of the keywords used in the program and their meaning can be found in Appendix 2. Forty-five (55.6%) of the soldiers sent the word SMOKED at least once, 31 (38.3%) sent the word STOP, and 30 (37.0%) sent the word NEED. Most of the soldiers (*n* = 69, 82.5%) sent at least one valid keyword, besides APPROVE, which was required in order to enroll. On average, each participant sent 7.6 ± 6.6 keywords based to usage data, similar to 6.6 ± 10.9 based on self-report. (Table 2).

**Table 2:** Participant interaction with the program, including keyword usage data (*N* = 81) and responses from survey (*N* = 63)

Participant interaction		n (%) or mean ± SD
Keyword usage data	NERVOUS	22 (27.2)
	BOREDOM	14 (17.3)
	SLIPPED_UP	11 (13.6)
	REASONS	17 (21.0)
	NEED	30 (37.0)
	CODE	3 (3.7)
	SMOKED	45 (55.6)
	STOP	31 (38.3)
	NEW	19 (23.5)
	QUIT	19 (23.5)
	FINAL	20 (24.7)
	Proportion of participants who sent more than 1 keyword (including non-valid)	74 (91.4)
	Proportion of participants who sent more than 1 valid keyword	69 (85.2)
	Total number of keywords sent by participant to system	7.6 ± 6.6
Self-report from survey	Answer to question: “how many times did you sent keywords?”	6.6 ± 10.9

*NA* not available/applicable, *SD* Standard deviation

Among participants that reported previous quit attempts, most of the participants thought the program was more helpful than other methods used previously (59.2%). Previous methods included several options with the most prevalent being: e-cigarettes (22%), self-help literature (16%), nicotine replacements (12%) and willpower (59%). Most participants reported reading “more than half of the messages”. Most participants stated “making an attempt” to quit (88.9%).

A small proportion of the participants reported having technical problems using the program (*n* = 5, 7.9%). Three participants reported having trouble choosing a new quit date. One participant, tried to send an open question to the system (despite this not being possible), and another did not understand how to send keywords. Most of the participants found the program easy to use (*n* = 61/63, 96.8%). The vast majority (84.1%), including many that had not quit smoking, stated that they would recommend the program to a friend. Only 66.7% agreed that the messages helped them quit. (Table 3).

**Table 3:** Survey responses on participant use of the program and their satisfaction (*N* = 63)

Question from survey	N (%)
Did you read most or all texts?	50 (79.4)
Did you encounter any problems during use of the program?	5 (7.9)
Did you make a real attempt to quit?	56 (88.9)
Did you change your quit date?	14 (22.2)
Did you leave the program?	12 (19.0)
Have you used the keywords?	38 (60.3)
Did you use the website?	9 (14.3)
Has program helped compared to previous quit attempts? (among 49 participants with previous attempts)	29 (59.2)
Was the program easy to use?	61 (96.8)
Did we send the right amount of messages a day?	42 (66.7)
Did the messages give you good ideas to help you quit?	44 (69.8)
Did the messages help you quit?	42 (66.7)
Did you feel that somebody cared if you quit?	45 (71.4)
Would you recommend the program to a friend?	53 (84.1)

Note: Outcomes in this table are the percentage that answered yes to the question

**Fig. 1:**
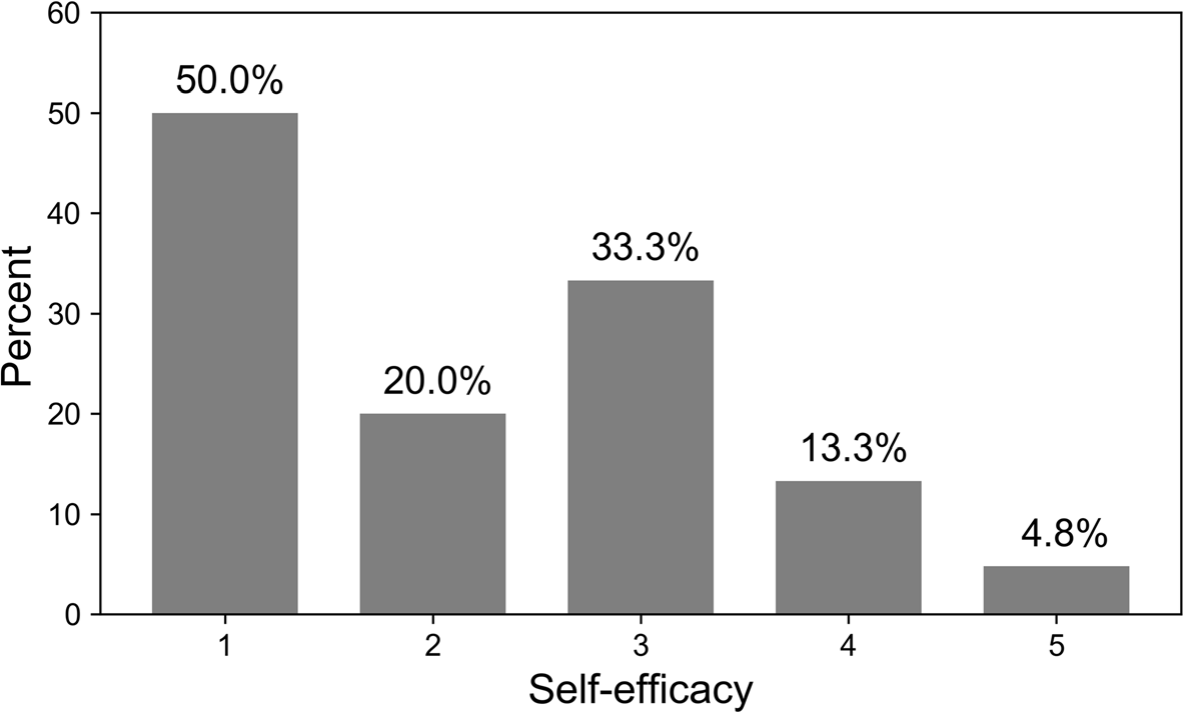
Chances (percentage) of leaving the program by self-efficacy as reported at baseline (1 = lowest efficacy)

Of the 63 responders, twelve (19.0%) reported leaving the program before completion. The only variable that was associated with chances of leaving the program was self-efficacy at baseline (mean = 2.9 ± 1.2 for those that quit the program and 3.9 ± 1.1 for those that did not, *p* = 0.011). For the lowest self-efficacy group the chances of leaving the program were 50.0%, while for the highest they were 4.8% (Fig. 1). Among men, 11/51 (21.6%) reported leaving the program, compared to 1/12 (8.3%) among women.

### Smoking outcomes

At 1 month follow up, 13 (20.6%) participants reported not smoking. Assuming all those lost to follow-up had returned to smoking, 16.0% were not smoking.

## Discussion

We evaluated for the first time the feasibility of a text-messaging smoking cessation program for soldiers in Israel. Recruitment of 81 participants was fast and efficient. The response rate for the online survey at 1 month was suboptimal (78%). Technical problems were rarely reported (7.9%). Participants’ engagement was high, as 82.5% sent a least one valid keyword and most participants reported reading more than half of the messages. Satisfaction was high as 96.8% found the program easy to use and 84.1% would recommend it to a friend. Low self-efficacy at baseline was associated with higher chances of leaving the program. At 1 month, 20.6% reported that they had not smoked in the past week. Overall, the study demonstrates that a text-messaging smoking cessation program is feasible in a military setting.

Engagement with the program was high, similar to the results seen in other studies [12, 16], again showing that text-based programs have the advantage of delivering the messages straight to the participant without requiring in-person engagement [19, 24]. The high engagement may be attributed to the soldiers being young and generally technologically savvy, as can be seen by their age distribution and ownership of smartphones.

The higher response rate to the online survey compared to the text-message survey raises questions about the best method of assessing such a program. Although a one-word answer could be thought to be easier for participants to respond to, this was not the case. One explanation may be the reminders that participants received for the online survey.

Participants that reported leaving the program had a significantly lower self-efficacy rate than those that remained in the program. Previous studies have shown that self-efficacy may predict smoking cessation [25–27], and although these results are preliminary, future studies should evaluate whether self-efficacy can predict program usability and smoking cessation.

Limitations of the study include a small sample size, and it not being a randomized control trial, meaning it was not designed to enable assessment of program effectiveness. We could not assess representativeness of study participants in comparison to the target population. We cannot rule out the possibility of self-selection into the study, as participants may be those that are more inclined to quit or are more technologically savvy than others. While the response to the survey was relatively high, it was not 100%, and it required a relatively large amount of effort from the research team, which would be problematic in a larger study.

Advantages of the study include it being based on previous successful programs [16] as well as it being the first study based on text-messaging both in Hebrew and in a military setting. Previous smoking cessation studies aimed for military populations, have shown feasibility using a booklet format [28]. Given that soldiers carry mobile phones while on duty, mobile technology may be a more promising format. However, some soldiers have limited access to their phones which may present a challenge for mobile-based programs. The advantage of having a smoking cessation program as a constant reminder in the participant’s pocket are minimized when access to the mobile phone is limited. It may be warranted to develop an alternate protocol for soldiers who have limited mobile phone access.

Smoking cessation programs in the military setting could be further supported smoke-free settings and policy. Commanders and peers can take advantage of the military authority and motivate soldiers to quit smoking. This is beginning to happen in the Israeli army, with new smoking restrictions being put into effect [29]. Lessons learned from the current study could aid in development of programs using newer technologies, such as smartphone applications, and in integration of the program with other smoking cessation and prevention strategies.

This study demonstrated advantages to adapting such a program to a specific group, and suggests that it may be worth designing a program to meet the needs of other specialized groups as well. Further thought should also be given to expanding this program to offer additional support during the program, while considering the challenges of a military setting. Text-messaging programs can be used as a stand-alone service or be used to supplement other services like group and phone consultation. Another feature to consider is the possibility of interaction between the participants themselves. Although this study provides evidence of feasibility, future large-scale studies will be needed in order to properly assess effectiveness of such programs, and integration with other smoking cessation programs.

## Conclusions

In conclusion, the study demonstrates that a text-messaging smoking cessation program is feasible in a military setting. Further development and evaluation of digital smoking cessation tools provided to soldiers are warranted.

## Appendix 1

**Table 4:** This table provides examples of some of the messages used during the program. Times stated in the negative represent the number of days before the chosen quit date, while positive numbers are the days starting from the quit date. If the participant chose fewer than 14 days, they received messages beginning on the appropriate day. For example, they began receiving messages for 1 week before cessation if they chose 7 days until quitting. All participants received the same “welcome messages” regardless of the number of days they chose

Timing	Message
− 13	Prescription medication is taken at least 8 days before quitting. Contact your unit’s physician today. The treatment must be purchased in a civilian pharmacy.
− 11	After quitting, the spots on your teeth and fingers will disappear, sexual performance will improve and you will generally feel much better.
−5	Tell family and friends in your unit that you are quitting and that their support is important to you. Make sure everybody knows. How about a Facebook status?
−1	Plan a goodbye ceremony from your final cigarette today. What will you do with it? How would you like to say goodbye? Suggestions in another message soon.
0	Congrats! You have reached the BIG day! We are right here with you, we know how much you want to quit and believe you will succeed.
3	Well done, you have passed 3 days without smoking! Your breathing is improving and it will continue to improve with each day that passes.
6	The days following quitting may be stressful. Try coping with physical activities, showering or relaxation techniques. You can also text: NERVOUS
9	Are your nerves getting to you? Don’t forget – we’re here at all hours. You can also text: NERVOUS
18	Every day that passes distances you from the bad habit of smoking. This is a great time to strengthen good habits such as exercise.
38	Try and think of things that you wanted in the past but decided they were too expensive. Maybe now that you aren’t smoking you can afford to get them.
58	Even if a friend tells you that he can smoke occasionally and not be addicted, don’t be tempted. Every time you smoke you’re at risk of going back to smoke.

## Appendix 2

**Table 5:** This table contains the keywords used during the program. The first seven keywords on this list were described on the support website, while the final four are keywords that could only be used when prompted by the system

Keyword	Explanation
NERVOUS	To be used when the participant is feeling annoyed, anxious or generally in a bad mood.
BOREDOM	To be used when the participant is bored.
SLIPPED UP	To be used when the participant has smoked at some time following her quit date. In Hebrew, this is a single word.
REASONS	To be used when the participant would like to be reminded of the reasons for quitting they listed during registration.
NEED	To be used when the participant is feeling the need to smoke.
CODE	To be used when the participant would like to be reminded the keywords on this list.
QUIT	To be used when the participant would like to leave the program.
APPROVE	After registration, the participant is required to reply APPROVE, in order to begin.
SMOKED	These are the responses available to periodic surveys regarding the participants’ smoking status. SMOKED means they are still smoking (or have quit and regressed), STOPPED means they have quit smoking.
STOPPED	
FINAL	The participant is required to reply FINAL after requesting to leave the program.
NEW	After answering SMOKED on a survey, participants are given one opportunity to restart the program and choose a new quit date.

## Appendix 3

**Table 6:** Registration survey: The registration survey was given in Hebrew

Category	Questions
Personal information	Name, religion, nickname (for program), birthday, e-mail, phone number, city
Smoking questions	Do you smoke at least once a day? How many cigarettes do you smoke per day? How many cigarettes have you smoked in the past week? How many times have you attempted to quit? Do you think you have the ability to quit? Have you used any of the following methods to attempt quitting? Do you live with a smoker? How long after waking up do you smoke your first cigarette? Is it difficult for you to abstain from smoking in places where smoking is banned? Which cigarette will you find the hardest to give up? During which hours of the day do you most frequently smoke? Do you smoke when sick in bed? Why do want to quit smoking?
Phone usage questions	Do you own a smartphone? Do you take your phone with you when you leave the house? Do you pay for each text message? How many text messages do you send per week? How many text messages do you receive per week? Have you previously registered to any text messaging services? What model phone do you own?
Socio-demographic questions	How did you hear about the program? What is your sex? Are you pregnant or planning to be pregnant in the next 6 months? Are you in military duty? Do you serve in an “open” or “closed” base? What is your relationship status? What is your birth country? What level are your reading and writing abilities in Hebrew?

## Abbreviations

aOR: Adjusted odds ratio
CI: Confidence interval
CPD: Cigarette per day
IDF: Israeli Defense Forces
NA: Not applicable/available
RR: Relative risk
SD: Standard deviation
